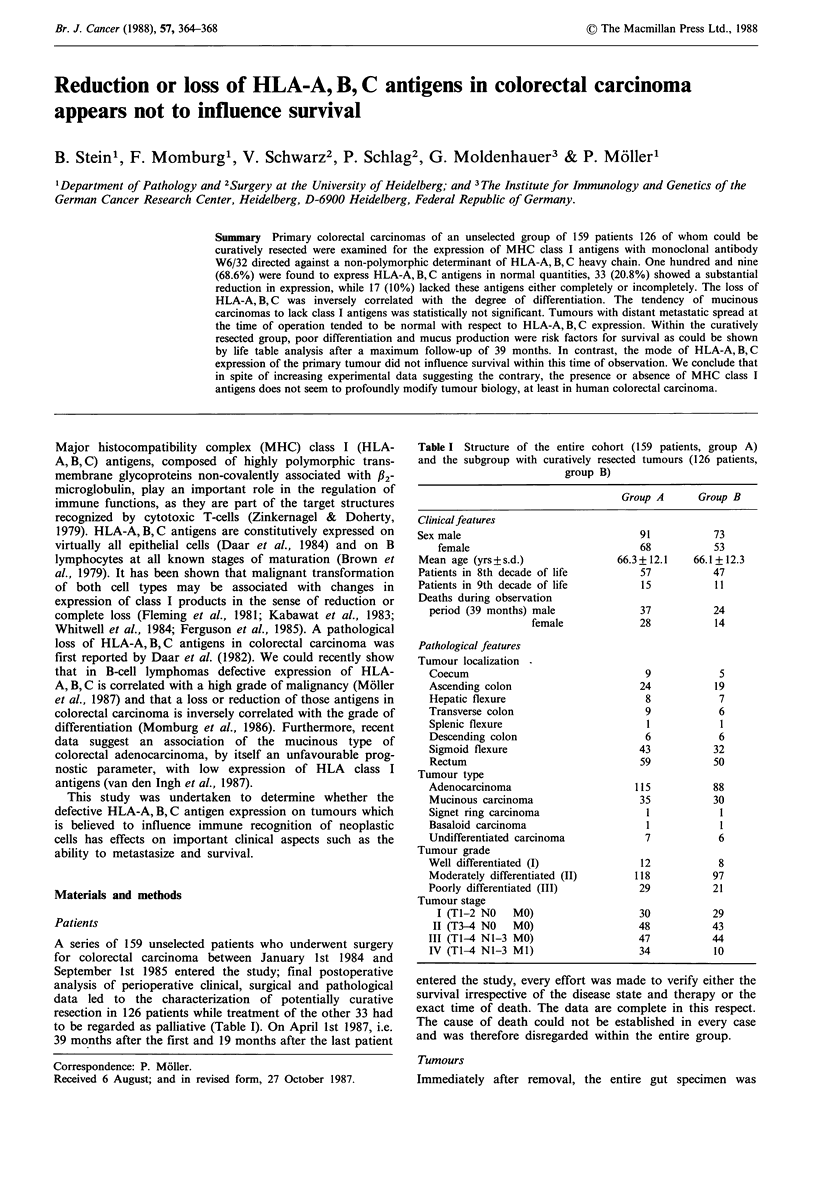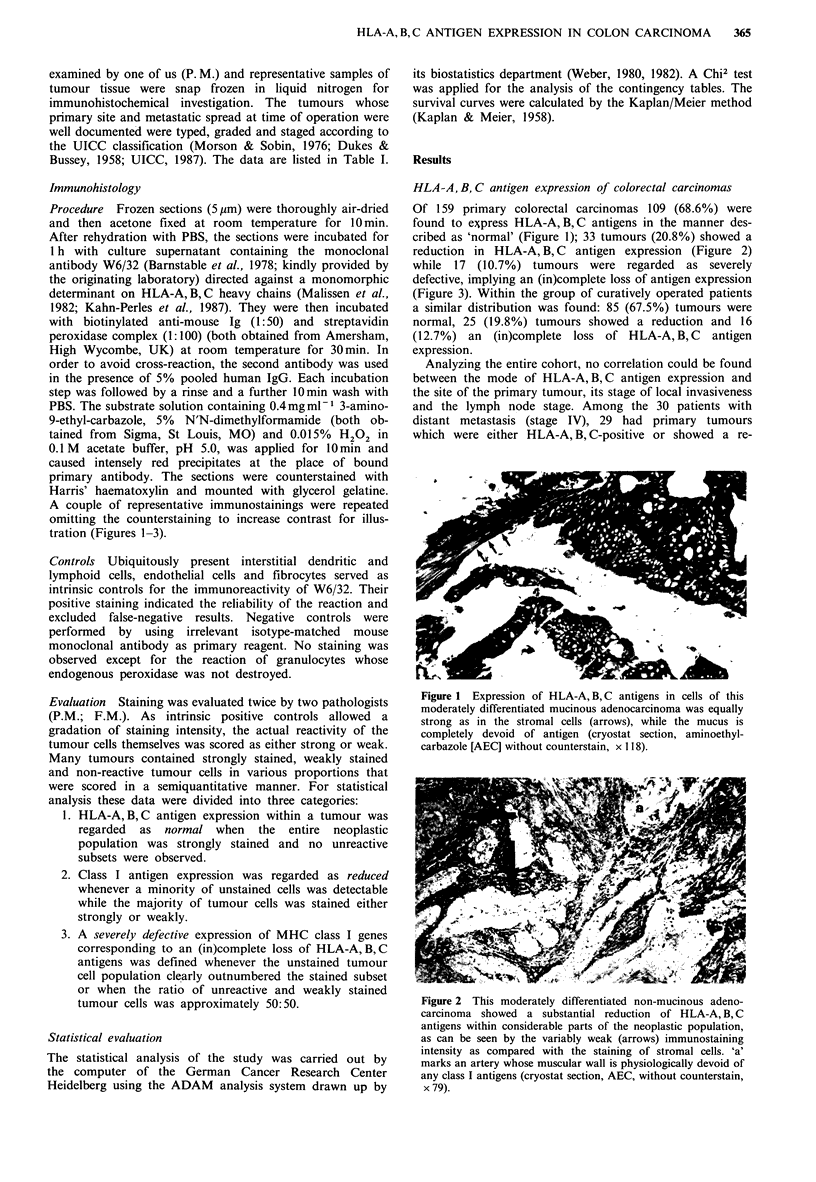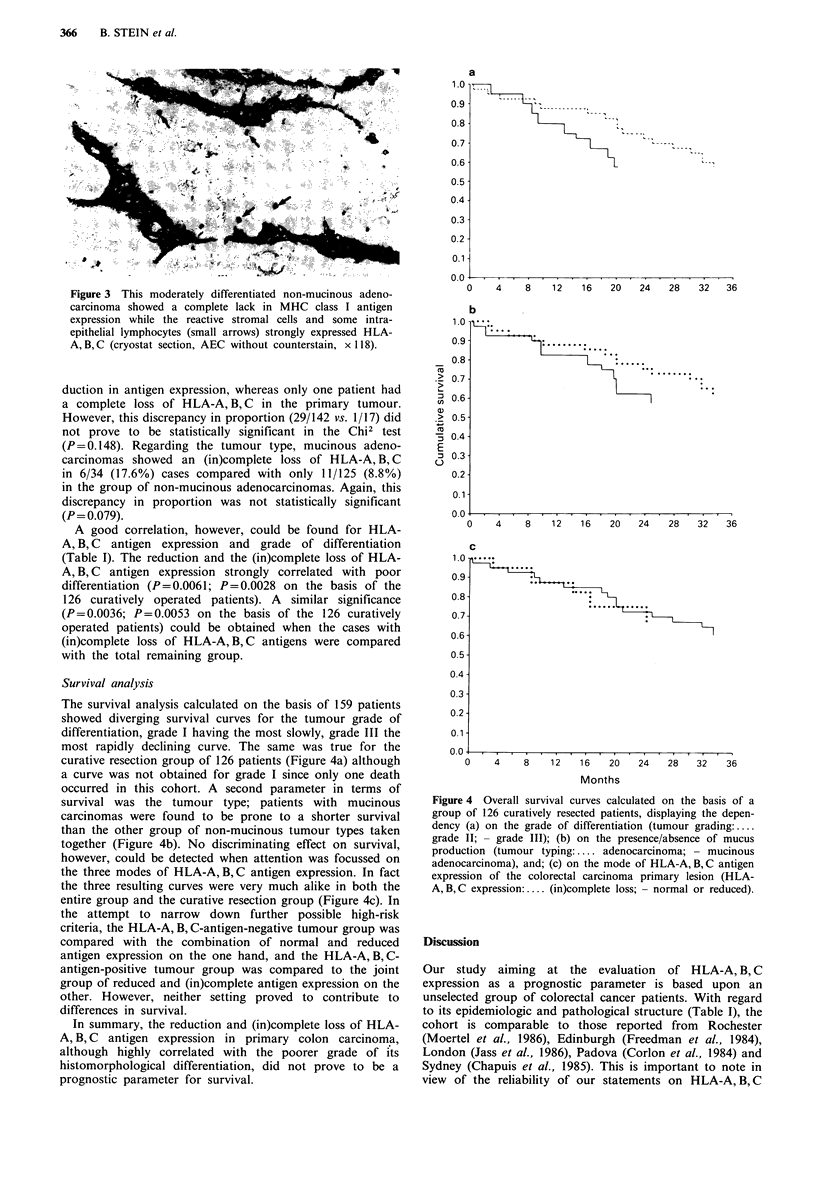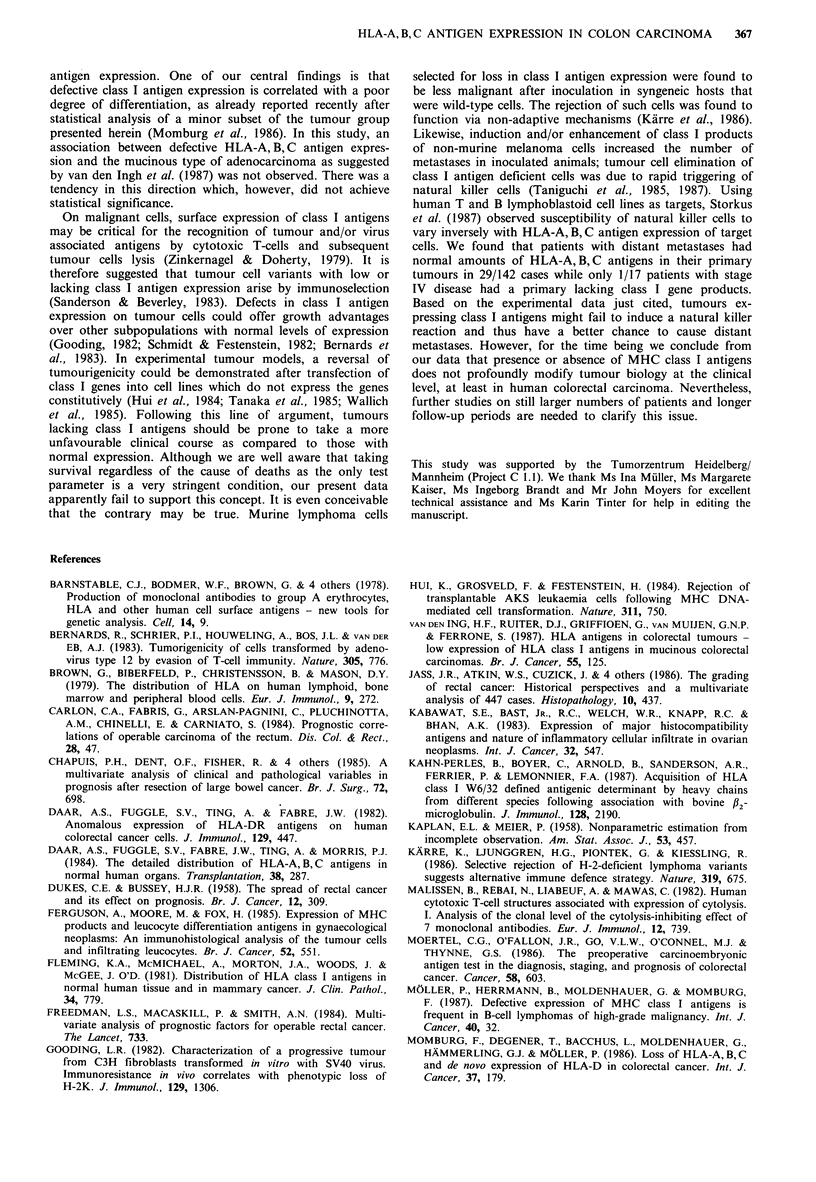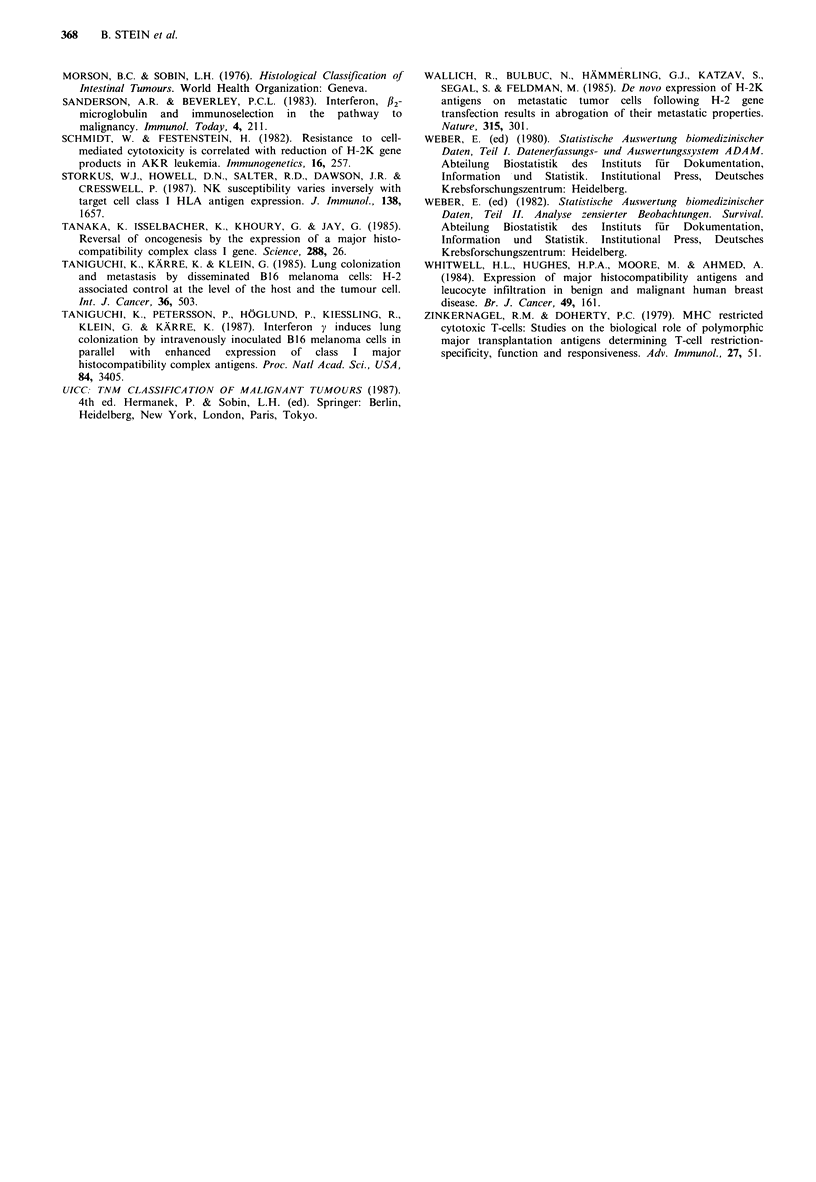# Reduction or loss of HLA-A,B,C antigens in colorectal carcinoma appears not to influence survival.

**DOI:** 10.1038/bjc.1988.83

**Published:** 1988-04

**Authors:** B. Stein, F. Momburg, V. Schwarz, P. Schlag, G. Moldenhauer, P. MÃ¶ller

**Affiliations:** Department of Pathology, University of Heidelberg, Federal Republic of Germany.

## Abstract

**Images:**


*Br. J. Cancer* (1988), **57**, 364–368 © The Macmillan Press Ltd., 1988

### Reduction or loss of HLA-A, B, C antigens in colorectal carcinoma appears not to influence survival

B. Stein[1], F. Momburg[1], V. Schwarz[2], P. Schlag[2], G. Moldenhauer[3] & P. Möller[1]

[1]*Department of Pathology and* [2]*Surgery at the University of Heidelberg; and* [3]*The Institute for Immunology and Genetics of the German Cancer Research Center, Heidelberg, D-6900 Heidelberg, Federal Republic of Germany.*

**Summary** Primary colorectal carcinomas of an unselected group of 159 patients 126 of whom could be curatively resected were examined for the expression of MHC class I antigens with monoclonal antibody W6/32 directed against a non-polymorphic determinant of HLA-A, B, C heavy chain. One hundred and nine (68.6%) were found to express HLA-A, B, C antigens in normal quantities, 33 (20.8%) showed a substantial reduction in expression, while 17 (10%) lacked these antigens either completely or incompletely. The loss of HLA-A, B, C was inversely correlated with the degree of differentiation. The tendency of mucinous carcinomas to lack class I antigens was statistically not significant. Tumours with distant metastatic spread at the time of operation tended to be normal with respect to HLA-A, B, C expression. Within the curatively resected group, poor differentiation and mucus production were risk factors for survival as could be shown by life table analysis after a maximum follow-up of 39 months. In contrast, the mode of HLA-A, B, C expression of the primary tumour did not influence survival within this time of observation. We conclude that in spite of increasing experimental data suggesting the contrary, the presence or absence of MHC class I antigens does not seem to profoundly modify tumour biology, at least in human colorectal carcinoma.

Major histocompatibility complex (MHC) class I (HLA-A, B, C) antigens, composed of highly polymorphic transmembrane glycoproteins non-covalently associated with $\beta_2$-microglobulin, play an important role in the regulation of immune functions, as they are part of the target structures recognized by cytotoxic T-cells (Zinkernagel & Doherty, 1979). HLA-A, B, C antigens are constitutively expressed on virtually all epithelial cells (Daar *et al.*, 1984) and on B lymphocytes at all known stages of maturation (Brown *et al.*, 1979). It has been shown that malignant transformation of both cell types may be associated with changes in expression of class I products in the sense of reduction or complete loss (Fleming *et al.*, 1981; Kabawat *et al.*, 1983; Whitwell *et al.*, 1984; Ferguson *et al.*, 1985). A pathological loss of HLA-A, B, C antigens in colorectal carcinoma was first reported by Daar *et al.* (1982). We could recently show that in B-cell lymphomas defective expression of HLA-A, B, C is correlated with a high grade of malignancy (Möller *et al.*, 1987) and that a loss or reduction of those antigens in colorectal carcinoma is inversely correlated with the grade of differentiation (Momburg *et al.*, 1986). Furthermore, recent data suggest an association of the mucinous type of colorectal adenocarcinoma, by itself an unfavourable prognostic parameter, with low expression of HLA class I antigens (van den Ingh *et al.*, 1987).

This study was undertaken to determine whether the defective HLA-A, B, C antigen expression on tumours which is believed to influence immune recognition of neoplastic cells has effects on important clinical aspects such as the ability to metastasize and survival.

### Materials and methods

### *Patients*

A series of 159 unselected patients who underwent surgery for colorectal carcinoma between January 1st 1984 and September 1st 1985 entered the study; final postoperative analysis of perioperative clinical, surgical and pathological data led to the characterization of potentially curative resection in 126 patients while treatment of the other 33 had to be regarded as palliative (Table I). On April 1st 1987, i.e. 39 months after the first and 19 months after the last patient entered the study, every effort was made to verify either the survival irrespective of the disease state and therapy or the exact time of death. The data are complete in this respect. The cause of death could not be established in every case and was therefore disregarded within the entire group.

**Table I** Structure of the entire cohort (159 patients, group A) and the subgroup with curatively resected tumours (126 patients, group B)

| | *Group A* | *Group B* |
|---|---|---|
| *Clinical features* | | |
| Sex male | 91 | 73 |
| female | 68 | 53 |
| Mean age (yrs ± s.d.) | 66.3 ± 12.1 | 66.1 ± 12.3 |
| Patients in 8th decade of life | 57 | 47 |
| Patients in 9th decade of life | 15 | 11 |
| Deaths during observation | | |
| period (39 months) male | 37 | 24 |
| female | 28 | 14 |
| *Pathological features* | | |
| Tumour localization | | |
| Coecum | 9 | 5 |
| Ascending colon | 24 | 19 |
| Hepatic flexure | 8 | 7 |
| Transverse colon | 9 | 6 |
| Splenic flexure | 1 | 1 |
| Descending colon | 6 | 6 |
| Sigmoid flexure | 43 | 32 |
| Rectum | 59 | 50 |
| Tumour type | | |
| Adenocarcinoma | 115 | 88 |
| Mucinous carcinoma | 35 | 30 |
| Signet ring carcinoma | 1 | 1 |
| Basaloid carcinoma | 1 | 1 |
| Undifferentiated carcinoma | 7 | 6 |
| Tumour grade | | |
| Well differentiated (I) | 12 | 8 |
| Moderately differentiated (II) | 118 | 97 |
| Poorly differentiated (III) | 29 | 21 |
| Tumour stage | | |
| I (T1–2 N0 M0) | 30 | 29 |
| II (T3–4 N0 M0) | 48 | 43 |
| III (T1–4 N1–3 M0) | 47 | 44 |
| IV (T1–4 N1–3 M1) | 34 | 10 |

### *Tumours*

Immediately after removal, the entire gut specimen was

Correspondence: P. Möller.
Received 6 August; and in revised form, 27 October 1987.

examined by one of us (P. M.) and representative samples of tumour tissue were snap frozen in liquid nitrogen for immunohistochemical investigation. The tumours whose primary site and metastatic spread at time of operation were well documented were typed, graded and staged according to the UICC classification (Morson & Sobin, 1976; Dukes & Bussey, 1958; UICC, 1987). The data are listed in Table I.

*Immunohistology*

*Procedure* Frozen sections (5 μm) were thoroughly air-dried and then acetone fixed at room temperature for 10 min. After rehydration with PBS, the sections were incubated for 1 h with culture supernatant containing the monoclonal antibody W6/32 (Barnstable *et al.*, 1978; kindly provided by the originating laboratory) directed against a monomorphic determinant on HLA-A, B, C heavy chains (Malissen *et al.*, 1982; Kahn-Perles *et al.*, 1987). They were then incubated with biotinylated anti-mouse Ig (1:50) and streptavidin peroxidase complex (1:100) (both obtained from Amersham, High Wycombe, UK) at room temperature for 30 min. In order to avoid cross-reaction, the second antibody was used in the presence of 5% pooled human IgG. Each incubation step was followed by a rinse and a further 10 min wash with PBS. The substrate solution containing 0.4 mg ml$^{-1}$ 3-amino-9-ethyl-carbazole, 5% N'N-dimethylformamide (both obtained from Sigma, St Louis, MO) and 0.015% $H_2O_2$ in 0.1 M acetate buffer, pH 5.0, was applied for 10 min and caused intensely red precipitates at the place of bound primary antibody. The sections were counterstained with Harris' haematoxylin and mounted with glycerol gelatine. A couple of representative immunostainings were repeated omitting the counterstaining to increase contrast for illustration (Figures 1–3).

*Controls* Ubiquitously present interstitial dendritic and lymphoid cells, endothelial cells and fibrocytes served as intrinsic controls for the immunoreactivity of W6/32. Their positive staining indicated the reliability of the reaction and excluded false-negative results. Negative controls were performed by using irrelevant isotype-matched mouse monoclonal antibody as primary reagent. No staining was observed except for the reaction of granulocytes whose endogenous peroxidase was not destroyed.

*Evaluation* Staining was evaluated twice by two pathologists (P.M.; F.M.). As intrinsic positive controls allowed a gradation of staining intensity, the actual reactivity of the tumour cells themselves was scored as either strong or weak. Many tumours contained strongly stained, weakly stained and non-reactive tumour cells in various proportions that were scored in a semiquantitative manner. For statistical analysis these data were divided into three categories:

1. HLA-A, B, C antigen expression within a tumour was regarded as *normal* when the entire neoplastic population was strongly stained and no unreactive subsets were observed.
2. Class I antigen expression was regarded as *reduced* whenever a minority of unstained cells was detectable while the majority of tumour cells was stained either strongly or weakly.
3. A *severely defective* expression of MHC class I genes corresponding to an (in)complete loss of HLA-A, B, C antigens was defined whenever the unstained tumour cell population clearly outnumbered the stained subset or when the ratio of unreactive and weakly stained tumour cells was approximately 50:50.

*Statistical evaluation*

The statistical analysis of the study was carried out by the computer of the German Cancer Research Center Heidelberg using the ADAM analysis system drawn up by its biostatistics department (Weber, 1980, 1982). A Chi$^2$ test was applied for the analysis of the contingency tables. The survival curves were calculated by the Kaplan/Meier method (Kaplan & Meier, 1958).

### Results

*HLA-A, B, C antigen expression of colorectal carcinomas*

Of 159 primary colorectal carcinomas 109 (68.6%) were found to express HLA-A, B, C antigens in the manner described as 'normal' (Figure 1); 33 tumours (20.8%) showed a reduction in HLA-A, B, C antigen expression (Figure 2) while 17 (10.7%) tumours were regarded as severely defective, implying an (in)complete loss of antigen expression (Figure 3). Within the group of curatively operated patients a similar distribution was found: 85 (67.5%) tumours were normal, 25 (19.8%) tumours showed a reduction and 16 (12.7%) an (in)complete loss of HLA-A, B, C antigen expression.

Analyzing the entire cohort, no correlation could be found between the mode of HLA-A, B, C antigen expression and the site of the primary tumour, its stage of local invasiveness and the lymph node stage. Among the 30 patients with distant metastasis (stage IV), 29 had primary tumours which were either HLA-A, B, C-positive or showed a re-

**Figure 1** Expression of HLA-A, B, C antigens in cells of this moderately differentiated mucinous adenocarcinoma was equally strong as in the stromal cells (arrows), while the mucus is completely devoid of antigen (cryostat section, aminoethylcarbazole [AEC] without counterstain, ×118).

**Figure 2** This moderately differentiated non-mucinous adenocarcinoma showed a substantial reduction of HLA-A, B, C antigens within considerable parts of the neoplastic population, as can be seen by the variably weak (arrows) immunostaining intensity as compared with the staining of stromal cells. 'a' marks an artery whose muscular wall is physiologically devoid of any class I antigens (cryostat section, AEC, without counterstain, ×79).

**Figure 3** This moderately differentiated non-mucinous adenocarcinoma showed a complete lack in MHC class I antigen expression while the reactive stromal cells and some intraepithelial lymphocytes (small arrows) strongly expressed HLA-A, B, C (cryostat section, AEC without counterstain, ×118).

duction in antigen expression, whereas only one patient had a complete loss of HLA-A, B, C in the primary tumour. However, this discrepancy in proportion (29/142 *vs.* 1/17) did not prove to be statistically significant in the $Chi^2$ test ($P=0.148$). Regarding the tumour type, mucinous adenocarcinomas showed an (in)complete loss of HLA-A, B, C in 6/34 (17.6%) cases compared with only 11/125 (8.8%) in the group of non-mucinous adenocarcinomas. Again, this discrepancy in proportion was not statistically significant ($P=0.079$).

A good correlation, however, could be found for HLA-A, B, C antigen expression and grade of differentiation (Table I). The reduction and the (in)complete loss of HLA-A, B, C antigen expression strongly correlated with poor differentiation ($P=0.0061$; $P=0.0028$ on the basis of the 126 curatively operated patients). A similar significance ($P=0.0036$; $P=0.0053$ on the basis of the 126 curatively operated patients) could be obtained when the cases with (in)complete loss of HLA-A, B, C antigens were compared with the total remaining group.

*Survival analysis*

The survival analysis calculated on the basis of 159 patients showed diverging survival curves for the tumour grade of differentiation, grade I having the most slowly, grade III the most rapidly declining curve. The same was true for the curative resection group of 126 patients (Figure 4a) although a curve was not obtained for grade I since only one death occurred in this cohort. A second parameter in terms of survival was the tumour type; patients with mucinous carcinomas were found to be prone to a shorter survival than the other group of non-mucinous tumour types taken together (Figure 4b). No discriminating effect on survival, however, could be detected when attention was focussed on the three modes of HLA-A, B, C antigen expression. In fact the three resulting curves were very much alike in both the entire group and the curative resection group (Figure 4c). In the attempt to narrow down further possible high-risk criteria, the HLA-A, B, C-antigen-negative tumour group was compared with the combination of normal and reduced antigen expression on the one hand, and the HLA-A, B, C-antigen-positive tumour group was compared to the joint group of reduced and (in)complete antigen expression on the other. However, neither setting proved to contribute to differences in survival.

In summary, the reduction and (in)complete loss of HLA-A, B, C antigen expression in primary colon carcinoma, although highly correlated with the poorer grade of its histomorphological differentiation, did not prove to be a prognostic parameter for survival.

**Figure 4** Overall survival curves calculated on the basis of a group of 126 curatively resected patients, displaying the dependency (a) on the grade of differentiation (tumour grading:.... grade II; – grade III); (b) on the presence/absence of mucus production (tumour typing:.... adenocarcinoma; – mucinous adenocarcinoma), and; (c) on the mode of HLA-A, B, C antigen expression of the colorectal carcinoma primary lesion (HLA-A, B, C expression:.... (in)complete loss; – normal or reduced).

### Discussion

Our study aiming at the evaluation of HLA-A, B, C expression as a prognostic parameter is based upon an unselected group of colorectal cancer patients. With regard to its epidemiologic and pathological structure (Table I), the cohort is comparable to those reported from Rochester (Moertel *et al.*, 1986), Edinburgh (Freedman *et al.*, 1984), London (Jass *et al.*, 1986), Padova (Corlon *et al.*, 1984) and Sydney (Chapuis *et al.*, 1985). This is important to note in view of the reliability of our statements on HLA-A, B, C

antigen expression. One of our central findings is that defective class I antigen expression is correlated with a poor degree of differentiation, as already reported recently after statistical analysis of a minor subset of the tumour group presented herein (Momburg *et al.*, 1986). In this study, an association between defective HLA-A, B, C antigen expression and the mucinous type of adenocarcinoma as suggested by van den Ingh *et al.* (1987) was not observed. There was a tendency in this direction which, however, did not achieve statistical significance.

On malignant cells, surface expression of class I antigens may be critical for the recognition of tumour and/or virus associated antigens by cytotoxic T-cells and subsequent tumour cells lysis (Zinkernagel & Doherty, 1979). It is therefore suggested that tumour cell variants with low or lacking class I antigen expression arise by immunoselection (Sanderson & Beverley, 1983). Defects in class I antigen expression on tumour cells could offer growth advantages over other subpopulations with normal levels of expression (Gooding, 1982; Schmidt & Festenstein, 1982; Bernards *et al.*, 1983). In experimental tumour models, a reversal of tumourigenicity could be demonstrated after transfection of class I genes into cell lines which do not express the genes constitutively (Hui *et al.*, 1984; Tanaka *et al.*, 1985; Wallich *et al.*, 1985). Following this line of argument, tumours lacking class I antigens should be prone to take a more unfavourable clinical course as compared to those with normal expression. Although we are well aware that taking survival regardless of the cause of deaths as the only test parameter is a very stringent condition, our present data apparently fail to support this concept. It is even conceivable that the contrary may be true. Murine lymphoma cells selected for loss in class I antigen expression were found to be less malignant after inoculation in syngeneic hosts that were wild-type cells. The rejection of such cells was found to function via non-adaptive mechanisms (Kärre *et al.*, 1986). Likewise, induction and/or enhancement of class I products of non-murine melanoma cells increased the number of metastases in inoculated animals; tumour cell elimination of class I antigen deficient cells was due to rapid triggering of natural killer cells (Taniguchi *et al.*, 1985, 1987). Using human T and B lymphoblastoid cell lines as targets, Storkus *et al.* (1987) observed susceptibility of natural killer cells to vary inversely with HLA-A, B, C antigen expression of target cells. We found that patients with distant metastases had normal amounts of HLA-A, B, C antigens in their primary tumours in 29/142 cases while only 1/17 patients with stage IV disease had a primary lacking class I gene products. Based on the experimental data just cited, tumours expressing class I antigens might fail to induce a natural killer reaction and thus have a better chance to cause distant metastases. However, for the time being we conclude from our data that presence or absence of MHC class I antigens does not profoundly modify tumour biology at the clinical level, at least in human colorectal carcinoma. Nevertheless, further studies on still larger numbers of patients and longer follow-up periods are needed to clarify this issue.

This study was supported by the Tumorzentrum Heidelberg/Mannheim (Project C 1.1). We thank Ms Ina Müller, Ms Margarete Kaiser, Ms Ingeborg Brandt and Mr John Moyers for excellent technical assistance and Ms Karin Tinter for help in editing the manuscript.

### References

BARNSTABLE, C.J., BODMER, W.F., BROWN, G. & 4 others (1978). Production of monoclonal antibodies to group A erythrocytes, HLA and other human cell surface antigens – new tools for genetic analysis. *Cell*, **14**, 9.

BERNARDS, R., SCHRIER, P.I., HOUWELING, A., BOS, J.L. & VAN DER EB, A.J. (1983). Tumorigenicity of cells transformed by adenovirus type 12 by evasion of T-cell immunity. *Nature*, **305**, 776.

BROWN, G., BIBERFELD, P., CHRISTENSSON, B. & MASON, D.Y. (1979). The distribution of HLA on human lymphoid, bone marrow and peripheral blood cells. *Eur. J. Immunol.*, **9**, 272.

CARLON, C.A., FABRIS, G., ARSLAN-PAGNINI, C., PLUCHINOTTA, A.M., CHINELLI, E. & CARNIATO, S. (1984). Prognostic correlations of operable carcinoma of the rectum. *Dis. Col. & Rect.*, **28**, 47.

CHAPUIS, P.H., DENT, O.F., FISHER, R. & 4 others (1985). A multivariate analysis of clinical and pathological variables in prognosis after resection of large bowel cancer. *Br. J. Surg.*, **72**, 698.

DAAR, A.S., FUGGLE, S.V., TING, A. & FABRE, J.W. (1982). Anomalous expression of HLA-DR antigens on human colorectal cancer cells. *J. Immunol.*, **129**, 447.

DAAR, A.S., FUGGLE, S.V., FABRE, J.W., TING, A. & MORRIS, P.J. (1984). The detailed distribution of HLA-A, B, C antigens in normal human organs. *Transplantation*, **38**, 287.

DUKES, C.E. & BUSSEY, H.J.R. (1958). The spread of rectal cancer and its effect on prognosis. *Br. J. Cancer*, **12**, 309.

FERGUSON, A., MOORE, M. & FOX, H. (1985). Expression of MHC products and leucocyte differentiation antigens in gynaecological neoplasms: An immunohistological analysis of the tumour cells and infiltrating leucocytes. *Br. J. Cancer*, **52**, 551.

FLEMING, K.A., McMICHAEL, A., MORTON, J.A., WOODS, J. & McGEE, J. O'D. (1981). Distribution of HLA class I antigens in normal human tissue and in mammary cancer. *J. Clin. Pathol.*, **34**, 779.

FREEDMAN, L.S., MACASKILL, P. & SMITH, A.N. (1984). Multivariate analysis of prognostic factors for operable rectal cancer. *The Lancet*, **733**.

GOODING, L.R. (1982). Characterization of a progressive tumour from C3H fibroblasts transformed *in vitro* with SV40 virus. Immunoresistance *in vivo* correlates with phenotypic loss of H-2K. *J. Immunol.*, **129**, 1306.

HUI, K., GROSVELD, F. & FESTENSTEIN, H. (1984). Rejection of transplantable AKS leukaemia cells following MHC DNA-mediated cell transformation. *Nature*, **311**, 750.

VAN DEN ING, H.F., RUITER, D.J., GRIFFIOEN, G., VAN MUIJEN, G.N.P. & FERRONE, S. (1987). HLA antigens in colorectal tumours – low expression of HLA class I antigens in mucinous colorectal carcinomas. *Br. J. Cancer*, **55**, 125.

JASS, J.R., ATKIN, W.S., CUZICK, J. & 4 others (1986). The grading of rectal cancer: Historical perspectives and a multivariate analysis of 447 cases. *Histopathology*, **10**, 437.

KABAWAT, S.E., BAST, JR., R.C., WELCH, W.R., KNAPP, R.C. & BHAN, A.K. (1983). Expression of major histocompatibility antigens and nature of inflammatory cellular infiltrate in ovarian neoplasms. *Int. J. Cancer*, **32**, 547.

KAHN-PERLES, B., BOYER, C., ARNOLD, B., SANDERSON, A.R., FERRIER, P. & LEMONNIER, F.A. (1987). Acquisition of HLA class I W6/32 defined antigenic determinant by heavy chains from different species following association with bovine $\beta_2$-microglobulin. *J. Immunol.*, **128**, 2190.

KAPLAN, E.L. & MEIER, P. (1958). Nonparametric estimation from incomplete observation. *Am. Stat. Assoc. J.*, **53**, 457.

KÄRRE, K., LJUNGGREN, H.G., PIONTEK, G. & KIESSLING, R. (1986). Selective rejection of H-2-deficient lymphoma variants suggests alternative immune defence strategy. *Nature*, **319**, 675.

MALISSEN, B., REBAI, N., LIABEUF, A. & MAWAS, C. (1982). Human cytotoxic T-cell structures associated with expression of cytolysis. I. Analysis of the clonal level of the cytolysis-inhibiting effect of 7 monoclonal antibodies. *Eur. J. Immunol.*, **12**, 739.

MOERTEL, C.G., O'FALLON, J.R., GO, V.L.W., O'CONNEL, M.J. & THYNNE, G.S. (1986). The preoperative carcinoembryonic antigen test in the diagnosis, staging, and prognosis of colorectal cancer. *Cancer*, **58**, 603.

MÖLLER, P., HERRMANN, B., MOLDENHAUER, G. & MOMBURG, F. (1987). Defective expression of MHC class I antigens is frequent in B-cell lymphomas of high-grade malignancy. *Int. J. Cancer*, **40**, 32.

MOMBURG, F., DEGENER, T., BACCHUS, L., MOLDENHAUER, G., HÄMMERLING, G.J. & MÖLLER, P. (1986). Loss of HLA-A, B, C and *de novo* expression of HLA-D in colorectal cancer. *Int. J. Cancer*, **37**, 179.

MORSON, B.C. & SOBIN, L.H. (1976). *Histological Classification of Intestinal Tumours.* World Health Organization: Geneva.

SANDERSON, A.R. & BEVERLEY, P.C.L. (1983). Interferon, $\beta_2$-microglobulin and immunoselection in the pathway to malignancy. *Immunol. Today,* **4,** 211.

SCHMIDT, W. & FESTENSTEIN, H. (1982). Resistance to cell-mediated cytotoxicity is correlated with reduction of H-2K gene products in AKR leukemia. *Immunogenetics,* **16,** 257.

STORKUS, W.J., HOWELL, D.N., SALTER, R.D., DAWSON, J.R. & CRESSWELL, P. (1987). NK susceptibility varies inversely with target cell class I HLA antigen expression. *J. Immunol.,* **138,** 1657.

TANAKA, K. ISSELBACHER, K., KHOURY, G. & JAY, G. (1985). Reversal of oncogenesis by the expression of a major histocompatibility complex class I gene. *Science,* **288,** 26.

TANIGUCHI, K., KÄRRE, K. & KLEIN, G. (1985). Lung colonization and metastasis by disseminated B16 melanoma cells: H-2 associated control at the level of the host and the tumour cell. *Int. J. Cancer,* **36,** 503.

TANIGUCHI, K., PETERSSON, P., HÖGLUND, P., KIESSLING, R., KLEIN, G. & KÄRRE, K. (1987). Interferon $\gamma$ induces lung colonization by intravenously inoculated B16 melanoma cells in parallel with enhanced expression of class I major histocompatibility complex antigens. *Proc. Natl Acad. Sci., USA,* **84,** 3405.

*UICC: TNM CLASSIFICATION OF MALIGNANT TUMOURS* (1987). 4th ed. Hermanek, P. & Sobin, L.H. (ed). Springer: Berlin, Heidelberg, New York, London, Paris, Tokyo.

WALLICH, R., BULBUC, N., HÄMMERLING, G.J., KATZAV, S., SEGAL, S. & FELDMAN, M. (1985). *De novo* expression of H-2K antigens on metastatic tumor cells following H-2 gene transfection results in abrogation of their metastatic properties. *Nature,* **315,** 301.

WEBER, E. (ed) (1980). *Statistische Auswertung biomedizinischer Daten, Teil I. Datenerfassungs- und Auswertungssystem ADAM.* Abteilung Biostatistik des Instituts für Dokumentation, Information und Statistik. Institutional Press, Deutsches Krebsforschungszentrum: Heidelberg.

WEBER, E. (ed) (1982). *Statistische Auswertung biomedizinischer Daten, Teil II. Analyse zensierter Beobachtungen. Survival.* Abteilung Biostatistik des Instituts für Dokumentation, Information und Statistik. Institutional Press, Deutsches Krebsforschungszentrum: Heidelberg.

WHITWELL, H.L., HUGHES, H.P.A., MOORE, M. & AHMED, A. (1984). Expression of major histocompatibility antigens and leucocyte infiltration in benign and malignant human breast disease. *Br. J. Cancer,* **49,** 161.

ZINKERNAGEL, R.M. & DOHERTY, P.C. (1979). MHC restricted cytotoxic T-cells: Studies on the biological role of polymorphic major transplantation antigens determining T-cell restriction-specificity, function and responsiveness. *Adv. Immunol.,* **27,** 51.